# Global research landscape of autoimmune nodopathy: a 20-year bibliometric analysis (2005–2025)

**DOI:** 10.1186/s13023-025-04091-7

**Published:** 2025-11-04

**Authors:** Mehmet Ertan Temir, Gizem Yavaş Temir

**Affiliations:** 1 Department of Clinical Neurophysiology, Neurology, Erzurum City Hospital, Yakutiye, Erzurum, Turkey; 2 Erzurum Provincial Health Directorate, Palandöken, Erzurum, Turkey

**Keywords:** Bibliyometric, Nodopathy, Autoimmune, Nodal, Paranodal

## Abstract

**Introduction:**

Autoimmune nodopathy (AN) is a recently recognized, rare immune-mediated neuropathy characterized by autoantibodies targeting nodal and paranodal proteins such as neurofascin-155 and contactin-1. Since its classification as a distinct entity in 2021 by European Academy of Neurology/Peripheral Nerve Society, scientific interest in AN has rapidly increased. This study aimed to conduct a comprehensive bibliometric analysis to identify research trends, influential publications, leading authors, institutions, and collaboration networks in the field of AN between 2005 and 2025.

**Methods:**

A literature search was performed in the Web of Science Core Collection using AN-related keywords. A total of 109 original English-language articles were included based on defined inclusion and exclusion criteria. VOSviewer and CiteSpace were used for network visualization and citation burst analysis. Descriptive statistics and trend projections were conducted using SPSS and Microsoft Excel.

**Results:**

A marked increase in publications was observed after 2021, coinciding with the formal definition of AN. The most productive countries were France, Germany, Spain, and Japan. Querol Luis, Sommer Claudia, and Doppler Kathrin emerged as leading authors. Highly cited articles focused on the pathogenesis and antibody profiles of AN. Keywords such as “neurofascin,” “IgG4,” and “autoimmune nodopathy” dominated the thematic clusters.

**Conclusion:**

This is first bibliometric analysis to systematically examine the scientific landscape of AN. Despite growing research interest, there remains a significant gap in large-scale epidemiological studies and randomized controlled trials. Our findings provide a roadmap for future research, highlighting the importance of international collaboration and antibody-based stratification in the diagnosis and treatment of AN.

## Introduction

Autoimmune nodopathy (AN) is a rare immune-mediated peripheral neuropathy [1]. Antibodies targeting nodal and paranodal proteins, such as contactin-1 (CNTN1), neurofascin-155 (NF155), contactin-associated protein 1 (CASPR1), as well as nodal-NF and pan-NF isoforms, are involved in the pathogenesis of the disease [2–4]. These antibodies interfere with connection between the terminal loops of the myelin sheath and axolemma, leading to significant conduction deficits without macrophage-mediated segmental demyelination [5, 6].

Acute-onset sensorimotor polyneuropathy, sensory ataxia, tremor, respiratory failure, cranial nerve involvement, nephrotic syndrome, strongly elevated CSF protein levels may be observed in AN [7–10]. Due to the subacute progressive onset, poor response to IVIG and corticosteroids and antibodies involved in its pathogenesis, it was started to be evaluated as a separate disease from Chronic Inflammatory Demyelinating Polyneuropathy (CIDP) variants by European Academy of Neurology/Peripheral Nerve Society (EAN/PNS) after 2021 [6]. Although the disease is more common in adults, reports of pediatric cases have increased in recent years [11, 12].

Current treatment options include IVIG, corticosteroids, rituximab, plasmapheresis, bortezomib, cyclophosphamide, azathioprine, methotrexate, and mycophenolate mofetil [11, 13–17]. Mortality rates vary between 20 and 50% in the literature. Especially Ig-1 pan-neurofascin has been associated with higher mortality [10, 18]. This represents a rare yet particularly severe phenotype, and therefore such rates should not be generalized to all AN case. To reduce morbidity and mortality due to AN, new treatments should be developed, antibodies should be detected with high sensitivity and specificity, and the pathogenesis should be fully elucidated.

Although the mechanisms of nodo-paranodopathy have been investigated for a long time, studies on this disease remain under the umbrella of CIDP since the definition of AN was not made until 2021 [19]. In addition, antibodies detected in AN are detected in less than 15% of all CIDP patients [20]. This shows that AN is a rare disease. It is very important for researchers to know the most influential articles and research trends for planning new research. Bibliometric analysis is a quantitative analysis method that allows to evaluate the impact of academic articles on a certain topic in the literature. In this analysis, current trends and gaps can be identified by examining many factors such as the most influential authors, countries, institutions, journals, publications. In this way, the general framework on the subject is drawn more easily and it is easier to plan new research by determining the areas to focus on [21].

Therefore, we aimed to perform a bibliometric analysis of original articles on AN that was available in the literature before the disease was defined and that was related to the disease. We tried to identify trends in topics, influential publications and authors, collaborations, and countries. In this way, we aim to provide a holistic view of AN research and a guide to finding new ideas and filling the gaps.

## Methods

The literature review was conducted by two independent researchers using the Web of Science Core Collection (WoSCC, Clarivate Analytics) database on 23.03.2025. Since the WoS database is mostly preferred in bibliometric studies, the search was performed in this database [21–25].

**Fig. 1:**
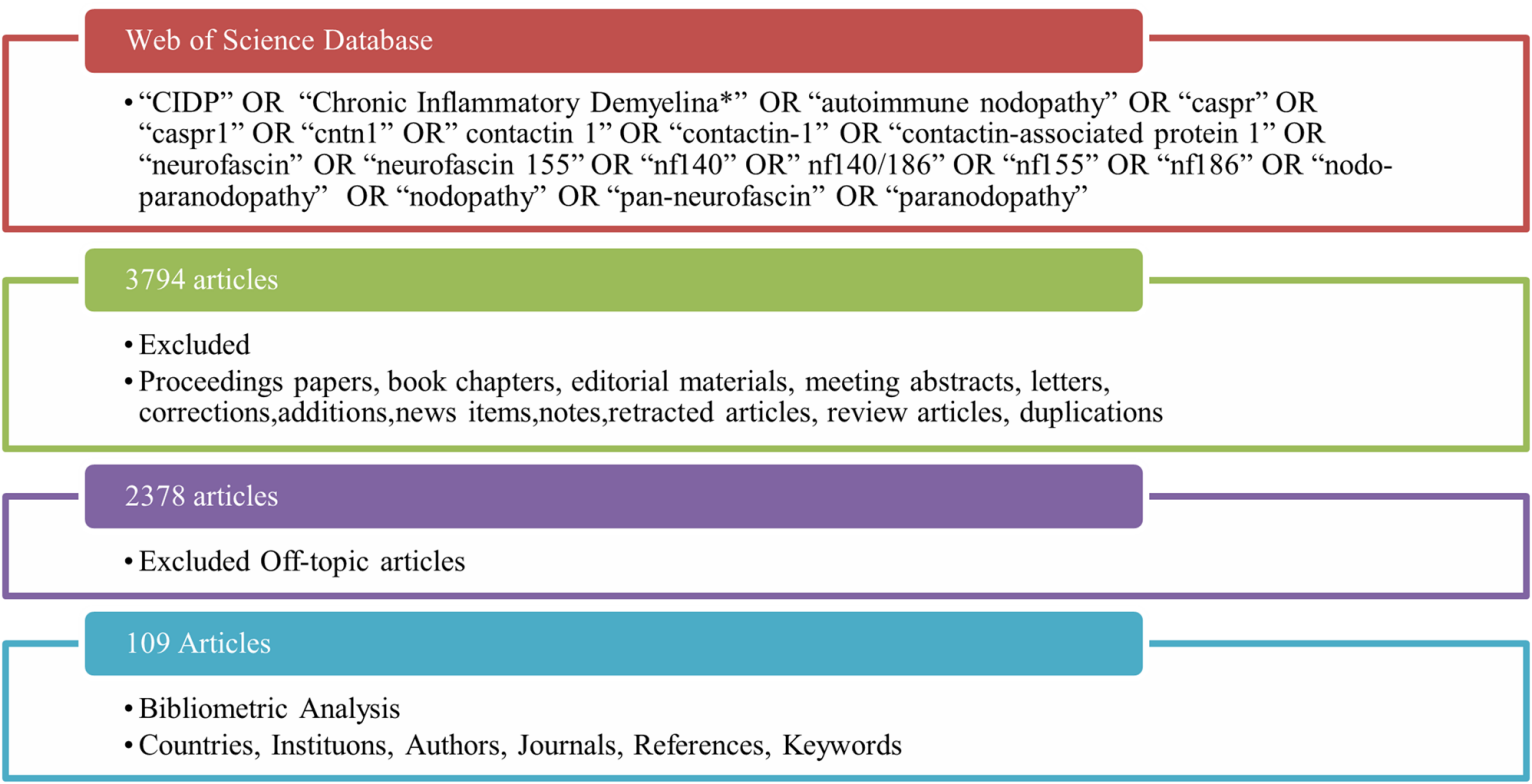
Flowchart of the study’s literature review strategy

‘CIDP’ OR ‘Chronic Inflammatory Demyelina*’ OR ‘autoimmune nodopathy’ OR ‘caspr’ OR ‘caspr1’ OR “cntn1” OR ‘contactin 1’ OR ‘contactin-1’ OR ‘contactin-associated protein 1’ OR ‘neurofascin’ OR ‘neurofascin 155’ OR ‘nf140’ OR ‘nf140/186’ OR ‘nf155’ OR ‘nf186’ OR ‘nodo-paranodopathy’ OR “nodopathy” OR ‘pan-neurofascin’ OR ‘paranodopathy’ keywords were used to access 3794 studies. Only original articles were included in the study. Proceedings papers, book chapters, editorial materials, meeting abstracts, letters, corrections, additions, news items, notes, retracted articles, review articles, duplications and off-topic articles were excluded. Since the EAN/PNS criteria were published in 2021, publications before this date were evaluated in terms of current AN diagnostic criteria and included in the study. Only English articles published between 2005 and 2025 were included in the study (Fig. 1). At the end of the filtering process, all information was recorded for the remaining 109 articles.

The VOSviewer package program (version 1.6.10) was used for bibliometric network visualization and analysis of these trend maps. [26–28]. In the map generated by VOSviewer, a node represents an object such as a country, institution, journal, and author. Node size and color indicate the number and classification of these items, respectively. The degree of collaboration or co-citation of items is reflected by the thickness of the lines between nodes [29, 30]. CiteSpace 6.4.R2 (Chaomei Chen, Drexel University, USA) was used to identify publications with high citation burst, keyword and reference analysis, and dual-map overlap analysis between journals. Thus, research trends in a particular topic were analyzed in more detail [28, 31]. Journal Citation Reports (JCR) 2023 was used to calculate the impact factor.

Microsoft Office Excel 2019 (Microsoft, Redmond, Washington, USA) was used to process the data and construct a linear regression model to predict the number of articles published in 2035. Data analysis was performed using SPSS (Statistical Package for the Social Sciences) version 25.0 software. A value of p < 0.05 was accepted as statistically significant. Linear regression analysis was used for the trend in the number of publications by year. An online bibliometric website (https://bibliometric.com/) was used to visualize the international collaboration between countries.

We applied the STROBE checklist.

Ethical considerations were not necessary for this study because it did not involve humans.

## Results

### Analysis of the number of publications and citations

A total of 3794 articles on AN in the WoSCC database were accessed using keywords. When exclusion criteria were applied, 2328 articles remained. Among these articles, those related to AN according to the EAN/PNS guideline were evaluated. At the last stage, 109 articles in English remained. These articles were published between 2007 and 2025. There were 106 articles in Science Citation Index Expanded (SCI-E) journals and three articles in Emerging Sources Citation Index (ESCI) journals.

**Fig. 2:**
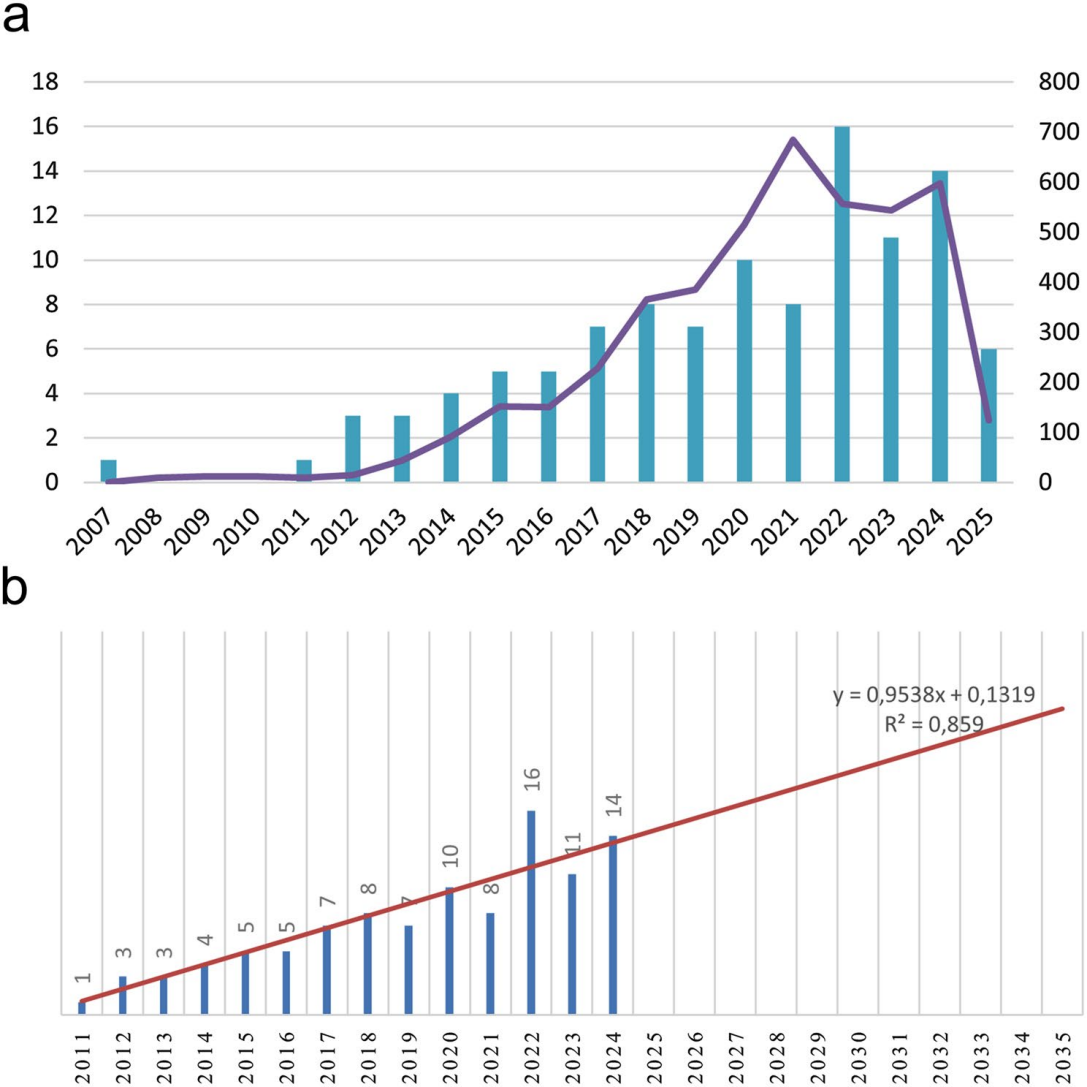
**a** number of publicitions and citations over the year. **b** projection of the trend in the number of publications per year

Among the 109 publications, most were retrospective and prospective clinical studies, followed by basic science/experimental investigations, with smaller numbers of neuropathology-based biopsy studies, case reports/series, and laboratory validation studies. Looking at the distribution of the number of publications by year, there was a significant increase in the number of articles, especially after 2021, due to the definition of AN. The year with the highest number of publications was 2022 with 16 articles. (Fig. 2a) In the linear regression analysis, a positive correlation was found between the number of publications per year 2011–2024 and the year of publication (R^2^ = 0.859, p < 0.001). Based on this model, the annual number of publications was predicted to be 28.74 in 2030 and 30.65 in 2032 (Fig. 2b). The cumulative number of citations per publication was 4494. The average number of citations per paper was 41.23.

### Analysis of contributions of prolific authors and co-cited authors

**Fig. 3:**
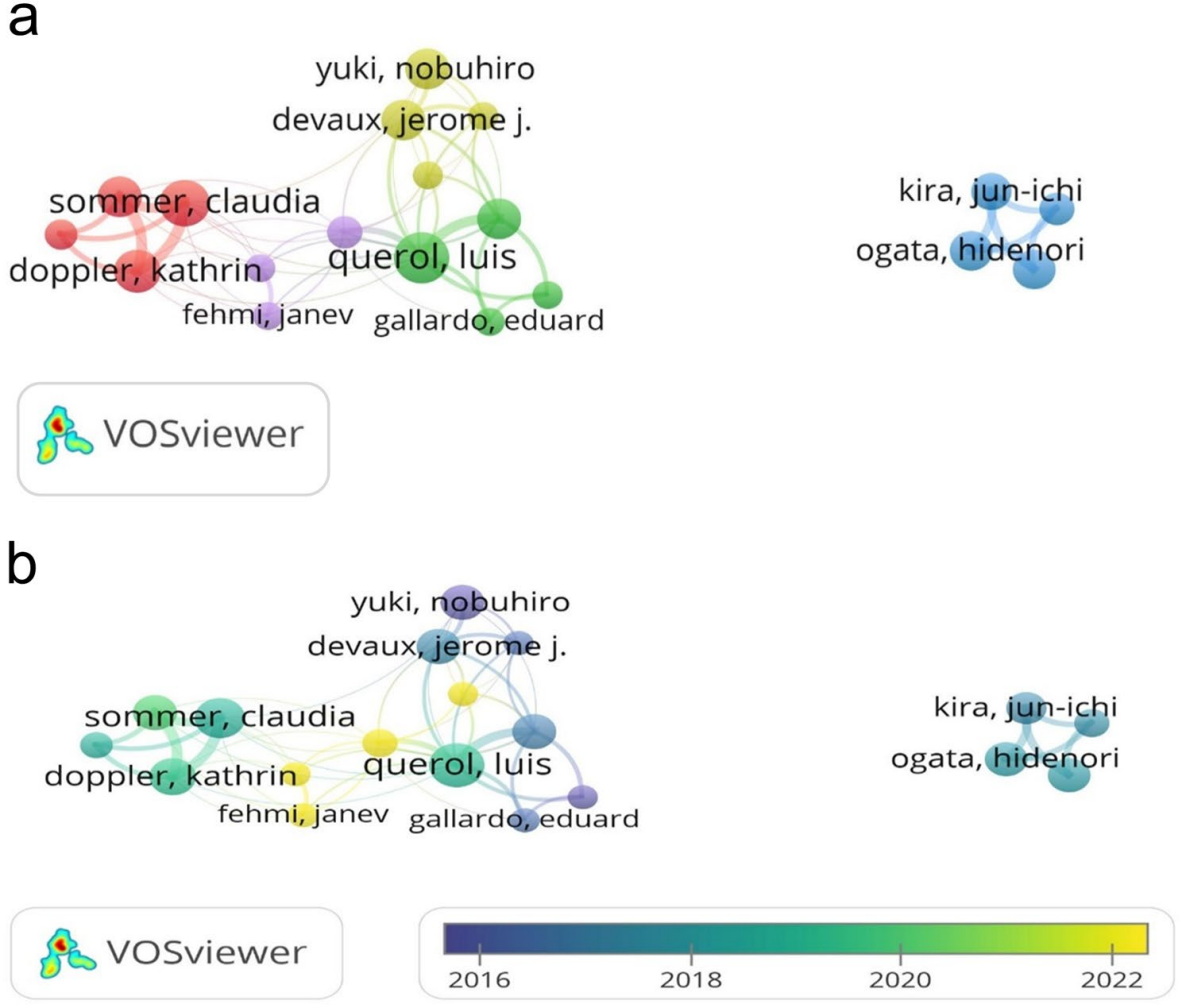
**a** Co-authorship network map. **b** Co-authorship overlay map

The most prolific authors on the topic were Querol, Luis, Sommer, Claudia and Doppler, Kathrin. The top 10 authors with the most publications and their respective citation criteria are shown in Table 1. When the co-authorship network was constructed among authors with more than five publications, five main clusters were identified in the resulting map. In this network analysis, the author with the highest number of links was Querol, Luis with 13 links (Fig. 3a). When the time-weighted (overlay) co-authorship map was analyzed, it was found that authors such as Lleixa, Cinta and Luise Appeltshauser have recently intensified their collaboration (Fig. 3b).

**Table 1:** The 10 authors with the most publications in the field of autoimmune nodopathy

Author	Country	Institution	Number of articles	C	CPP
Querol, Luis	SPAIN	Universitat Autònoma de BarcelonaUniversitat Autònoma de Barcelona	21	4,230	19.77
Sommer, Claudia	GERMANY	University of Würzburg	17	23,331	39,67
Doppler, Kathrin	GERMANY	University of Würzburg	15	2,210	20,4
Appeltshauser, Luise	GERMANY	University of Würzburg	13	533	20.97
Illa, Isabel	SPAIN	Universitat Autònoma de BarcelonaUniversitat Autònoma de Barcelona	13	15,524	45.13
Devaux, jerome	FRANCE	Universite de Montpellier	12	2,987	50,23
Yuki, Nobuhiro	JAPAN	Takai Hospital	12	2,833	14.35
Ogata, Hidenori	JAPAN	Kyushu University	10	1,030	33.3
Yamasaki, Ryo	JAPAN	Kyushu University	10	7,014	28.91
Lleixa, Cinta	SPAİN	Universitat Autònoma de BarcelonaUniversitat Autònoma de Barcelona	9	434	9.28

C: Citations

CCP: Citations per publication

### Analysis of contributions by institutions

**Fig. 4:**
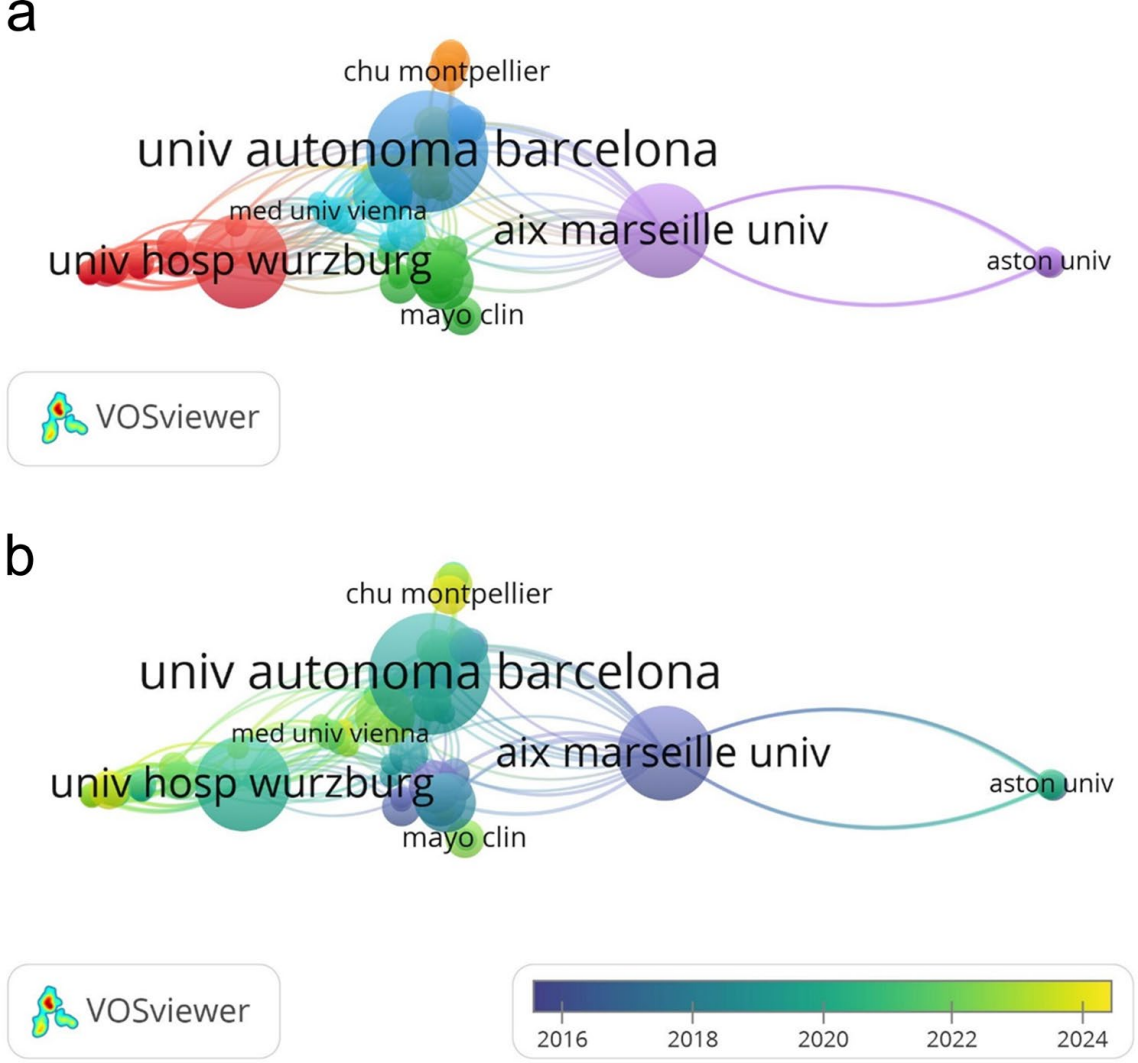
**a** Instittional Co-authorship network Map. **b** institutional Co-authorship overlay Map

When analyzed on an institutional basis, Hospital of Santa Creu I Sant Pau, Universitat Autònoma de BarcelonaUniversitat Autònoma de Barcelona, Aix Marseille University, Centre National De La Recherche Scientifique Cnrs, University of Würzburg, Institut National De La Sante Et De La Recherche Medicale Inserm, Universite De Montpellier were the institutions with the highest number of publications. The analysis of the network of institutional co-authorship was performed using a minimum number of publications of two. A network structure of 91 institutions and 10 clusters was obtained. The institutional co-authorship network resulting from this analysis and the network and overlay map with time distribution maps are shown in Fig. 4a and b.

### Analysis of the contributions of countries

**Fig. 5:**
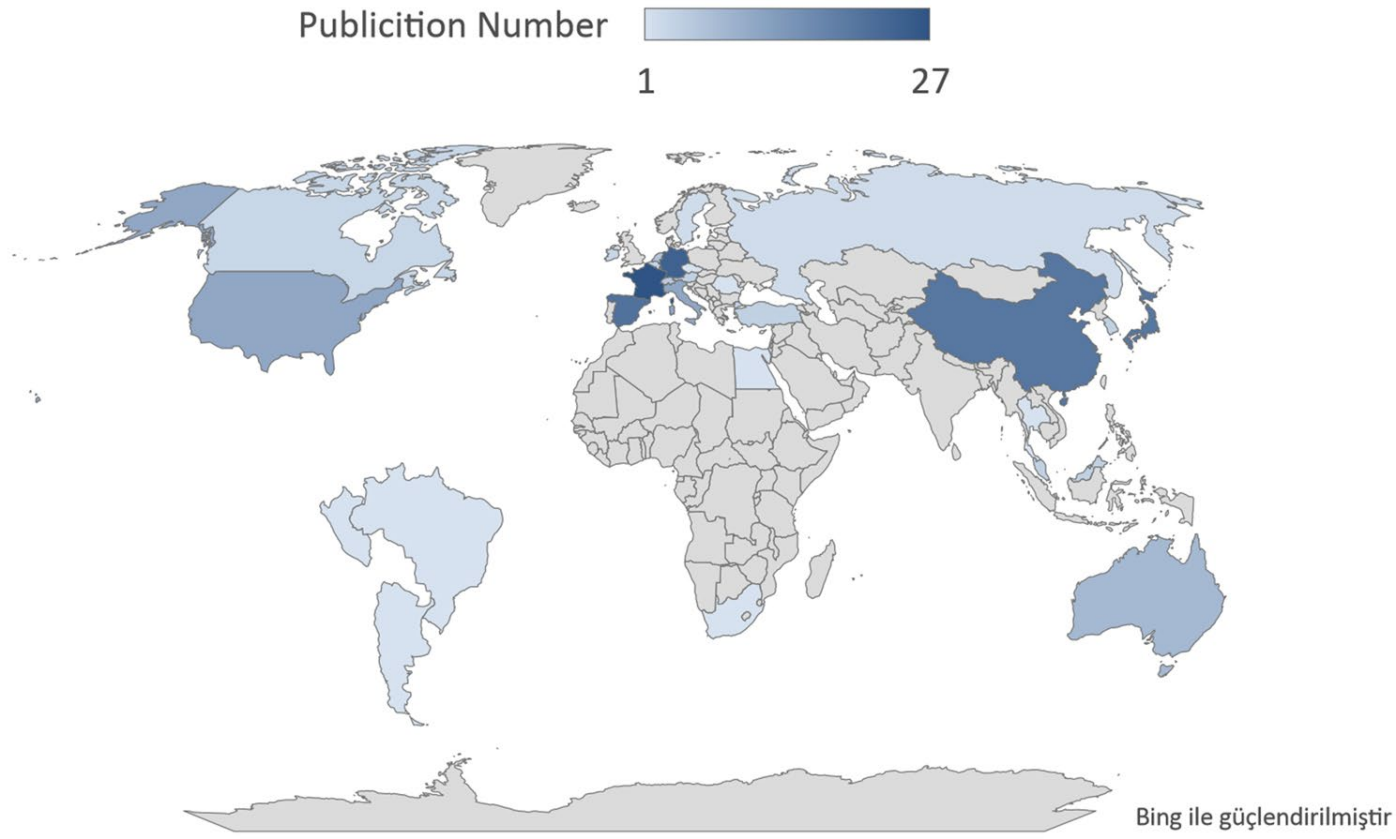
Geographic distribution of global publications on autoimmune nodopathy

**Fig. 6:**
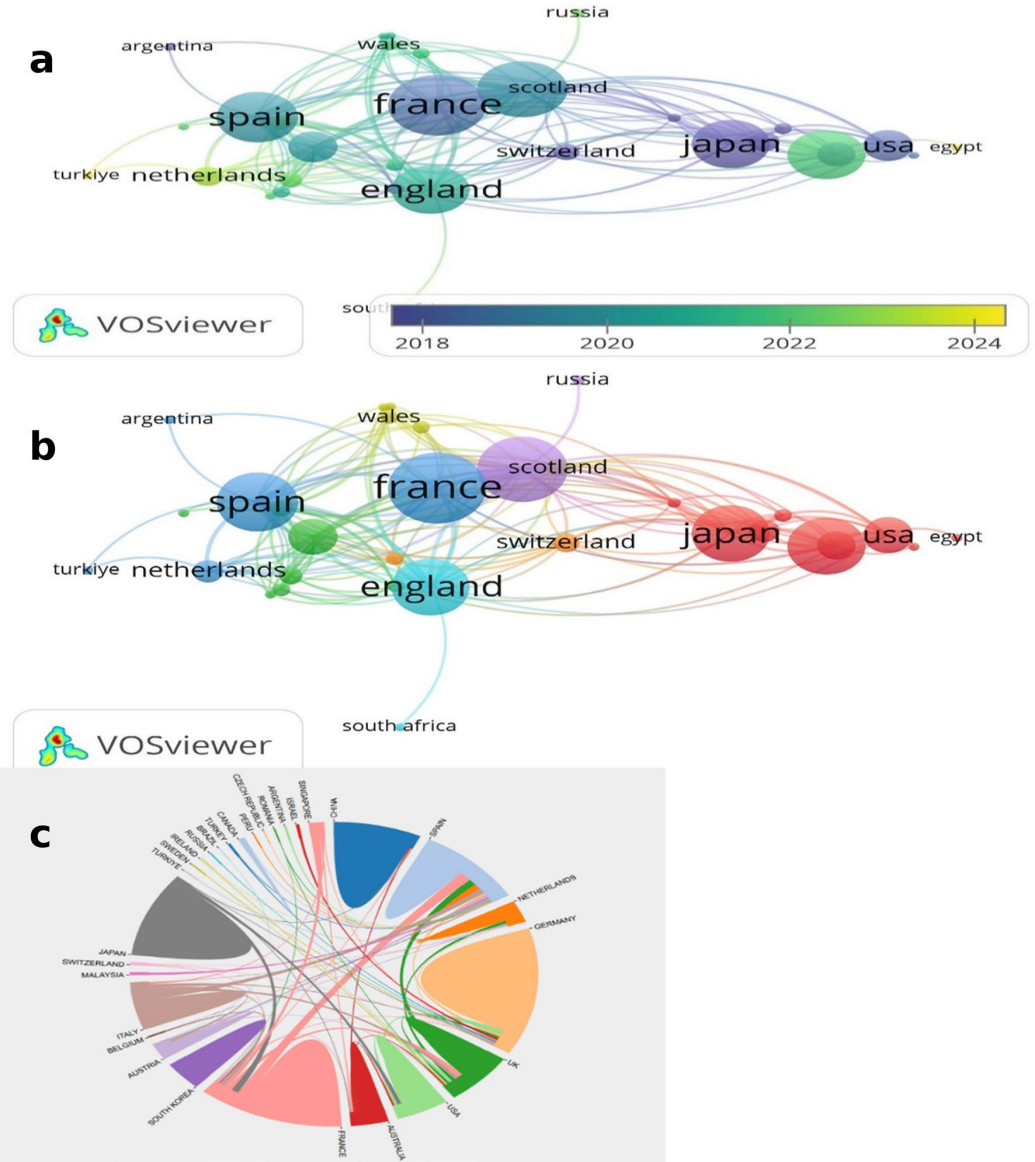
**a** Co-authorship overlay map by country. **b** Co-authorship network map by country. **c** the international collaborations’ visualization map of countries

The number of publications from a total of 32 countries with publications on the topic is shown on the geographical world map. (Fig. 5) The countries with the highest number of publications were France, Germany and Spain, respectively. The overlay and network maps of the network and time distribution maps of author collaborations by country are shown in Fig. 6a and b. In the cross-country collaboration map, particularly strong interactions between European countries were found (Fig. 6c).

### Analysis of the contributions of journals

**Fig. 7:**
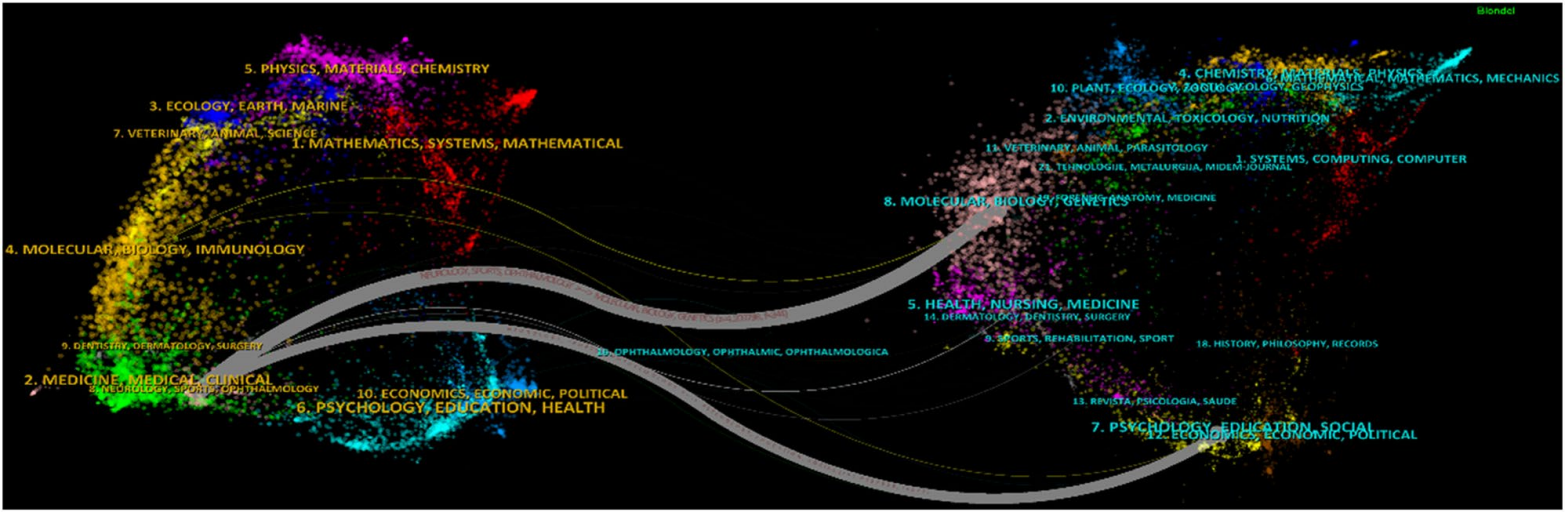
The dual-map overlay of journals

The 109 articles identified on AN were published in a total of 40 different journals. The most prolific journals were Neurology Neuroimmunology & Neuroinflammation, Frontiers in Immunology, Journal of The Peripheral Nervous System. The top 10 journals with the most publications are shown in Table 2. The result of the dual-map overlay analysis performed to examine the subject distribution of AN publications by journal is shown in Fig. 7. In this map, citing journals are on the left side of the map and cited journals are on the right side. The labels on the map represent the scientific disciplines covered by the journals. The coloured lines extending from left to right show which fields the citations are directed from which fields. It was observed that there were two distinct citation paths. Accordingly, it was observed that studies in Neurology/Sports/Ophthalmology journals frequently cited studies in Molecular/Biology/Genetics and Psychology/Education/Social journals.

**Table 2:** Top 10 journals with the most publications

Journal Name	Number of Articles	Journal Impact Factor 2023	Journal Citation Indicator 2023
NEUROLOGY NEUROIMMUNOLOGY& NEUROINFLAMMATION	14	8.3	2.42
FRONTIERS IN IMMUNOLOGY	8	5.7	0.95
JOURNAL OF THE PERIPHERAL NERVOUS SYSTEM	8	4	0.9
ANNALS OF CLINICAL AND TRANSLATIONAL NEUROLOGY	6	4.4	1.36
BRAIN	6	11.9	3.35
JOURNAL OF NEUROIMMUNOLOGY	6	2.9	0.61
JOURNAL OF NEUROLOGY NEUROSURGERY AND PSYCHIATRY	6	8.8	2.68
NEUROLOGY	6	8.4	2.55
JOURNAL OF NEUROLOGY	5	4.8	1.33
EUROPEAN JOURNAL OF NEUROLOGY	3	4.5	1.3

### Analysis of a highly cited studies

**Fig. 8:**
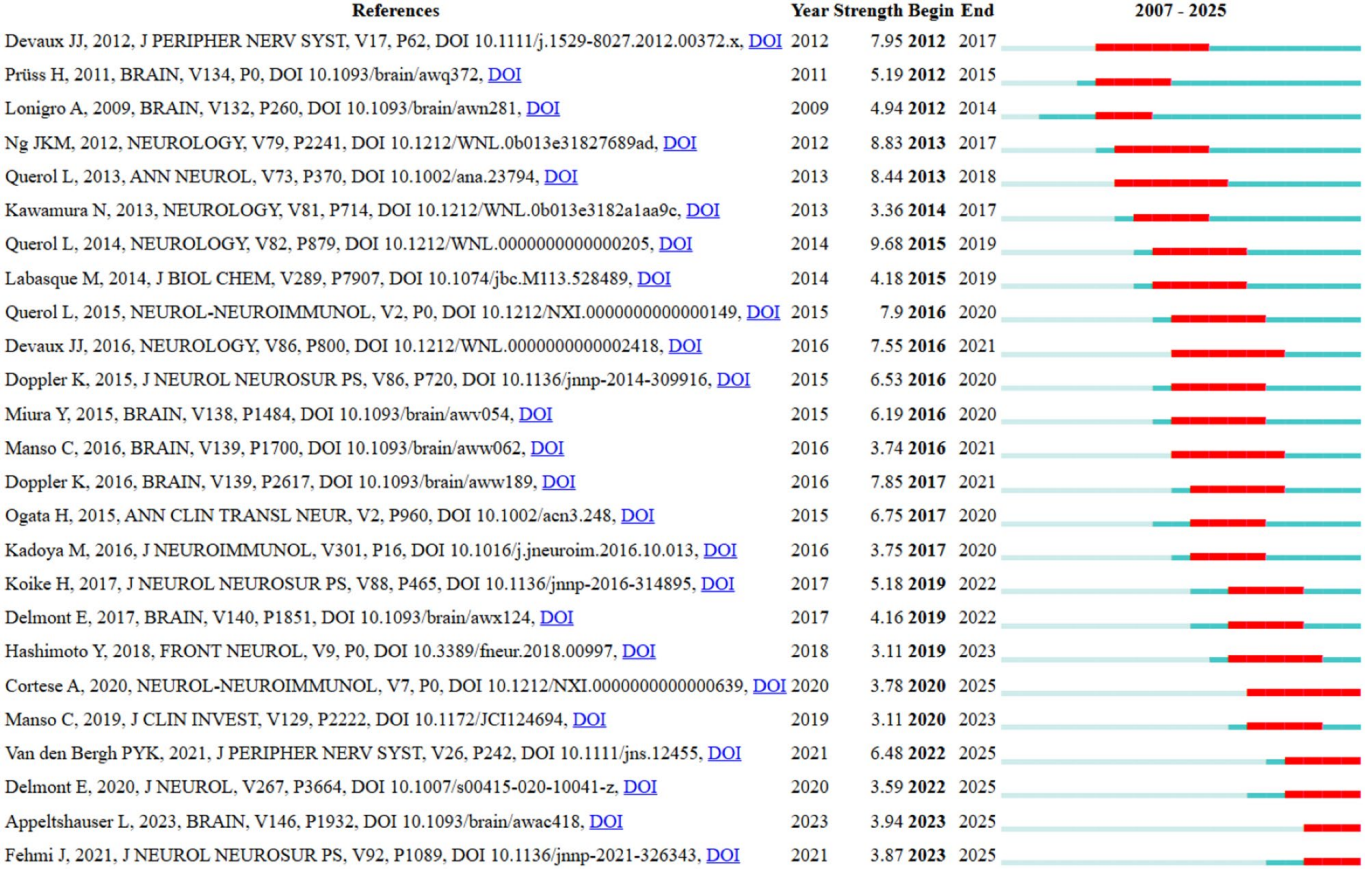
Top 25 references with the strongest citation bursts

The most cited article was ‘Neurofascin IgG4 antibodies in CIDP associate with disabling tremor and poor response to IVIg’ by Querol, Luis et al. with 265 citations. This paper was also the paper with the strongest citation burst between 2015 and 2019. When focusing on study types in the top 10 most cited articles, six were retrospective clinical studies, one was a prospective therapeutic case series, two were basic science experimental studies, and one was a neuropathology-based biopsy study. This indicates that, while experimental work is frequent overall, the studies with the highest citation impact have been primarily clinical investigations on antibody characterization and patient stratification. Information on the 10 most cited papers is in Table 3. The analysis of the top 25 papers showing a strongest citation burst in specific periods is in Fig. 8.

**Table 3:** Top 10 most cited articles

Rank	Article Title	Authors	Institution		Journal	Year	Cites	Cites per Year	Study Type
**1.**	Neurofascin IgG4 antibodies in CIDP associate with disabling tremor and poor response to IVIg	Querol, Luis et al.	Universitat Autònoma de BarcelonaUniversitat Autònoma de Barcelona	NEUROLOGY		2014	265	22,08	Retrospective observational clinical
**2.**	Antibodies to contactin-1 in chronic inflammatory demyelinating polyneuropathy	Querol, Luis et al.	Universitat Autònoma de BarcelonaUniversitat Autònoma de Barcelona		ANNALS OF NEUROLOGY	2013	257	19,77	Retrospective observational clinical
**3.**	Neurofascin-155 IgG4 in chronic inflammatory demyelinating polyneuropathy	Devaux, Jerome J et al.	Aix-Marseille Universite		NEUROLOGY	2016	196	19,6	Retrospective observational clinical
**4.**	Neurofascin as a target for autoantibodies in peripheral neuropathies	Ng, Judy King Man et al.	University of Munich		NEUROLOGY	2012	186	13,29	Mixed: retrospective observational + experimental
**5.**	Rituximab in treatment-resistant CIDP with antibodies against paranodal proteins	Querol, Luis et al.	Universitat Autònoma de BarcelonaUniversitat Autònoma de Barcelona		NEUROLOGY-NEUROIMMUNOLOGY & NEUROINFLAMMATION	2015	184	16,73	Prospective therapeutic case series
**6.**	Gangliosides contribute to stability of paranodal junctions and ion channel clusters in myelinated nerve fibers	Susuki, Keiichiro et al.	University of Connecticut		GLIA	2007	161	8,47	Experimental basic science
**7.**	Autoantibodies to nodal isoforms of neurofascin in chronic inflammatory demyelinating polyneuropathy	Delmont, Emilien et al.	Aix-Marseille Universite		BRAIN	2017	155	17,22	Retrospective observational clinical
**8.**	Paranodal dissection in chronic inflammatory demyelinating polyneuropathy with anti-neurofascin-155 and anti-contactin-1 antibodies	Koike, Haruki et al.	Nagoya University		JOURNAL OF NEUROLOGY NEUROSURGERY AND PSYCHIATRY	2017	148	16,44	Neuropathology-based
**9.**	Nodal proteins are target antigens in Guillain-Barre syndrome	Devaux, Jerome J et al.	Aix-Marseille Universite		JOURNAL OF THE PERIPHERAL NERVOUS SYSTEM	2012	148	10,57	Retrospective observational clinical + in vitro assays
**10.**	Characterization of IgG4 anti-neurofascin 155 antibody-positive polyneuropathy	Ogata, Hidenori et al.	Kyushu University		ANNALS OF CLINICAL AND TRANSLATIONAL NEUROLOGY	2015	143	13	Retrospective observational clinical

### Analysis of keywords

**Fig. 9:**
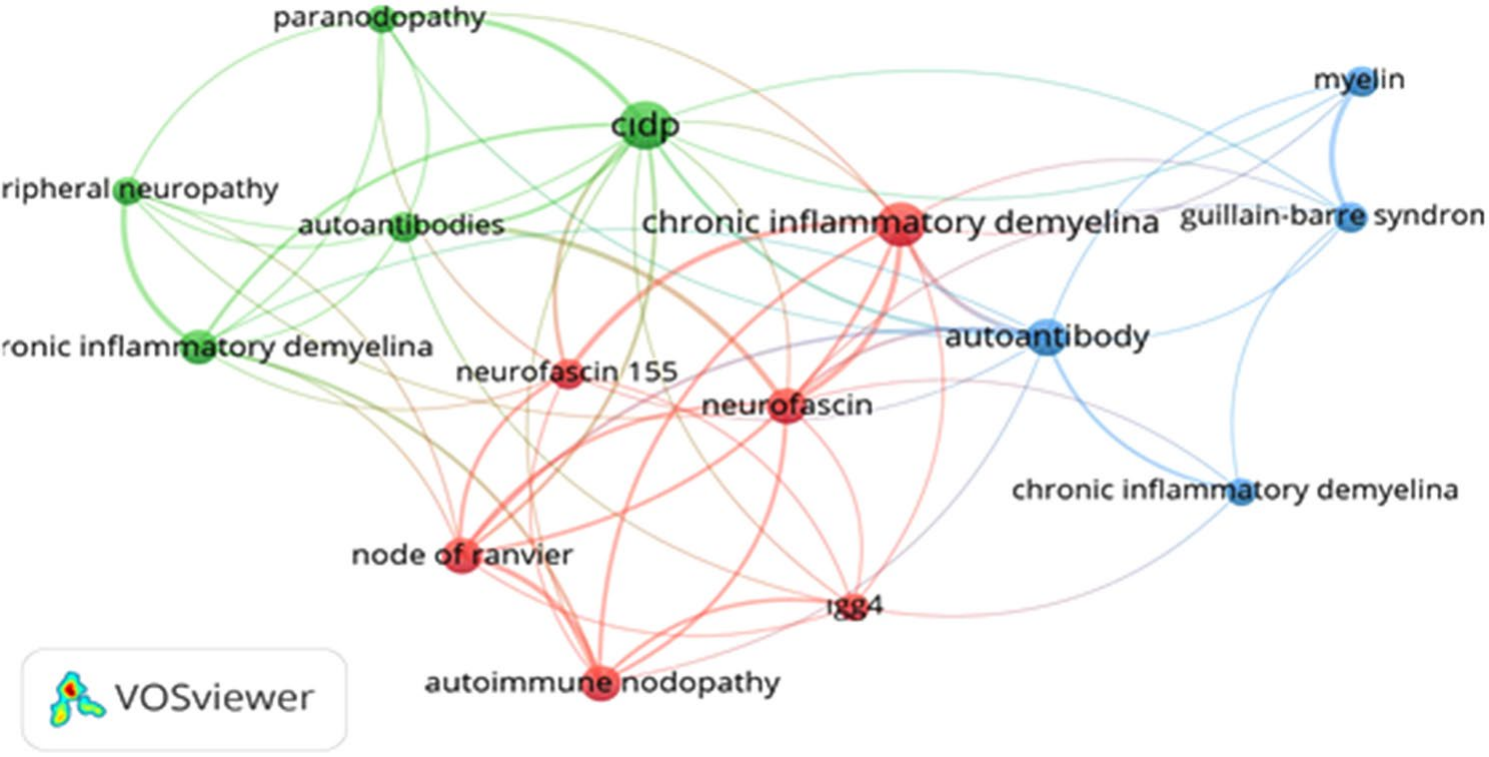
Network visualization map of top 15 keywords

Among a total of 209 keywords, the most used ones were ‘CIDP’, ‘Chronic inflammatory demyelinating polyneuropathy’, ‘autoantibody’, ‘neurofascin’, ‘autoimmune nodopathy’, and the most used keywords in the most cited articles were ‘ganglioside’, ‘gullian-barre syndrome’, “myelin”, ‘autoimmune neuropathy’, ‘axo-glial junction’. The network map of 15 keywords used more than 5% is shown in Fig. 9. These keywords are divided into 3 clusters. Cluster 1 (red); autoimmune nodopathy, ‘Chronic inflammatory demyelinating polyneuropathy’, ‘IgG4’, “neurofascin”, ‘neurofascin 155’, ‘node of ranvier’, Cluster 2 (green); ‘autoantibodies’, ‘Chronic inflammatory demyelinating polyradiculoneuropathy’, ‘CIDP’, ‘paranodopathy’, ‘peripheral neuropathy’, Cluster 3(blue); “autoantibody”, ‘Chronic inflammatory demyelinating’, ‘gullian-barre syndrome’, ‘myelin’. In the keyword citation burst analysis, the five keywords with the strongest citation burst were ‘nerve society guideline’, ‘neuropathy’, “ranvier”, ‘autoimmune nodopathy’, ‘polyneuropathy’. The keyword with the highest citation burst is ‘nerve society guideline’ with 4.78 between 2022 and 2025. ‘autoimmune nodopathy’ had a citation burst strength of 3.04 between 2022 and 2025.

### Timeline and conceptual cluster analysis

According to the cluster data obtained in the timeline view, the changes in certain thematic areas of AN research over time can be seen. (Fig. 1^10^) Accordingly, ‘contactin-associated protein’, ‘pan-neurofascin antibodies’, ‘peripheral nerve antigen’, ‘chronic inflammatory demyelinating polyneuropathy’, ‘paranodal protein’ are more recently used subject areas.

**Fig. 10:**
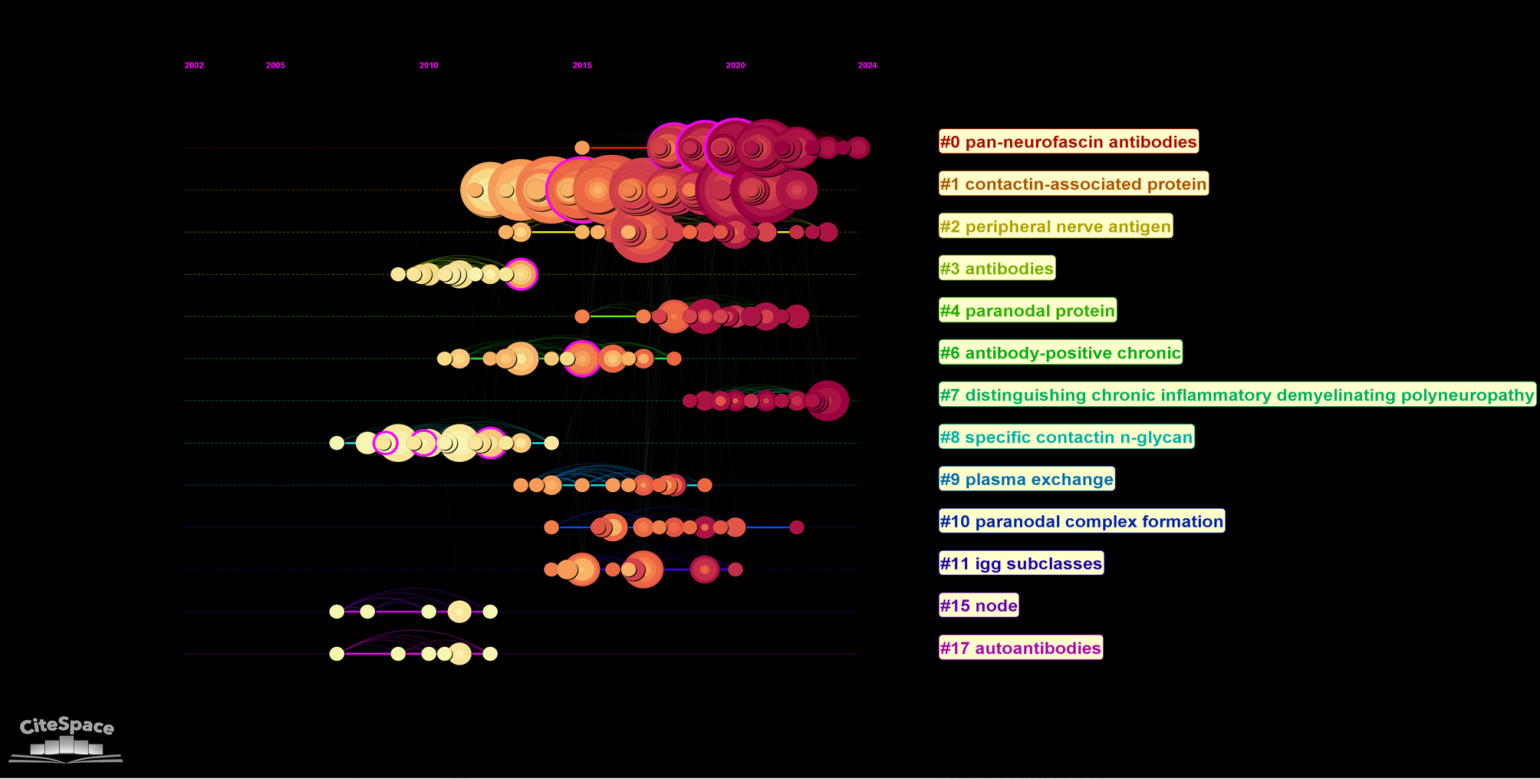
Timeline and keyword clustering display for the AN

### Analysis of Co-cited references

**Fig. 11:**
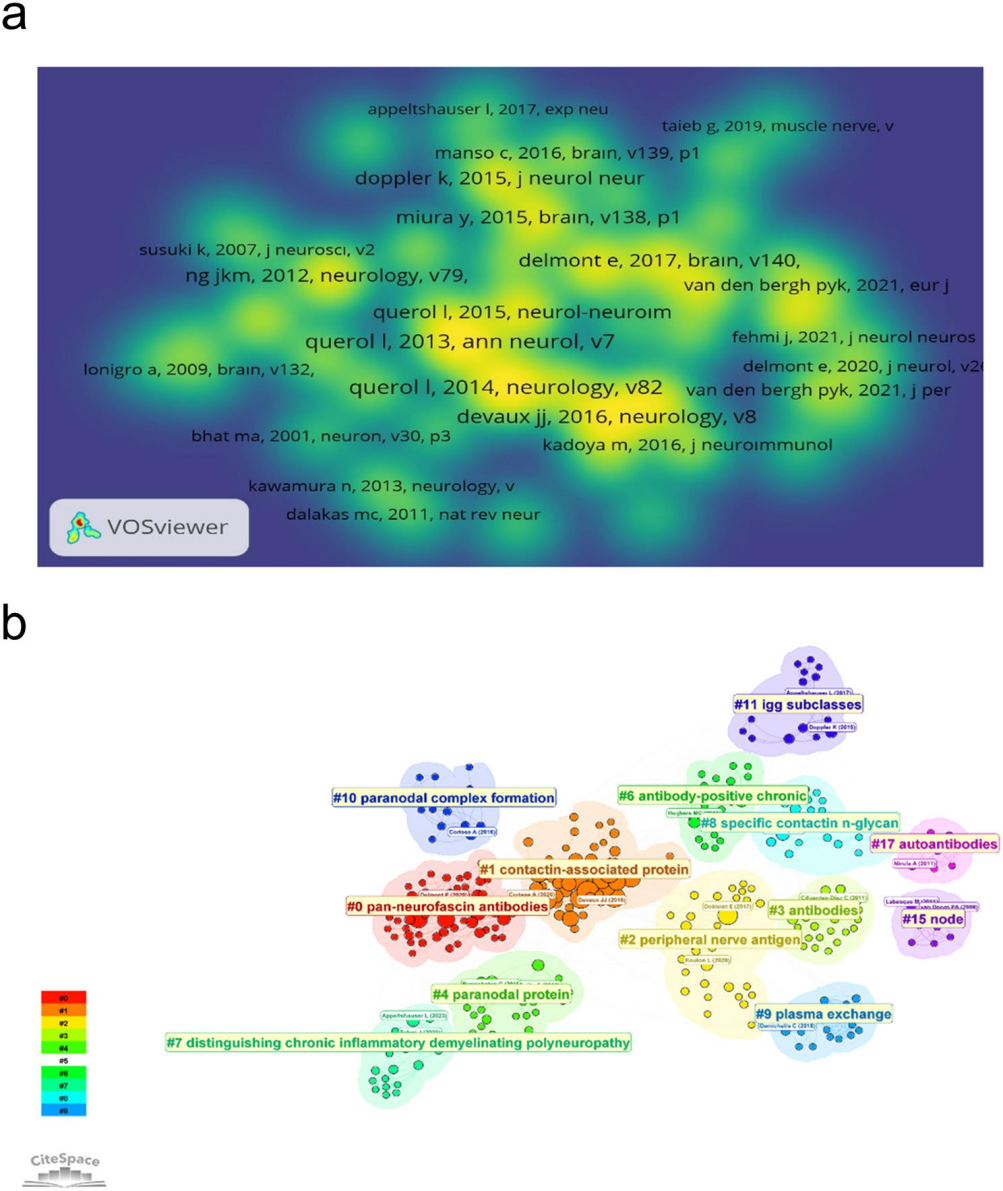
**a** density visualization map of the top 49 cited articles. **b** Co-cited reference network and cluster

The top 10 most co-cited references in the co-citation analysis are summarised in Table 4. CiteSpace was used to construct the network of co-cited references and cluster analysis revealed 13 clusters (Figure 11b). Modularity Q (0.741) and Mean Silhouette (0.869) values were both greater than 0.7. The first cluster label on the map was ‘pan-neurofascin antibodies’ and the second cluster label was ‘contactin-associated protein’. The co-citation density map of 49 articles with more than 10 cited articles out of a total of 1709 cited references is shown in Fig. 11a.

**Table 4:** Top 10 Co-cited references

Rank	Article Title	Authors	Institution	Journal	Year	Co-Cititations	Total Link Strength
1.	Neurofascin IgG4 antibodies in CIDP associate with disabling tremor and poor response to IVIg	Querol, Luis et al.	Universitat Autònoma de BarcelonaUniversitat Autònoma de Barcelona	NEUROLOGY	2014	63	531
2.	Antibodies to contactin-1 in chronic inflammatory demyelinating polyneuropathy	Querol, Luis et al.	Universitat Autònoma de BarcelonaUniversitat Autònoma de Barcelona	ANNALS OF NEUROLOGY	2013	61	518
3.	Neurofascin-155 IgG4 in chronic inflammatory demyelinating polyneuropathy	Devaux, Jerome J et al.	Aix-Marseille Universite	NEUROLOGY	2016	51	446
4	Characterization of IgG4 anti-neurofascin 155 antibody-positive polyneuropathy	Ogata, Hidenori et al.	Kyushu University	ANNALS OF CLINICAL AND TRANSLATIONAL NEUROLOGY	2015	43	397
5	Auto-antibodies to contactin-associated protein 1 (Caspr) in two patients with painful inflammatory neuropathy	Doppler, Kathrin et al.	University of Würzburg	BRAIN	2016	42	393
6	Contactin 1 IgG4 associates to chronic inflammatory demyelinating polyneuropathy with sensory ataxia	Miura, Yumako et al.	National University of Singapore	BRAIN	2015	40	390
7	Destruction of paranodal architecture in inflammatory neuropathy with anti-contactin-1 autoantibodies	Doppler, Kathrin et al.	University of Würzburg	J NEURO	2015	39	363
8.	Neurofascin as a target for autoantibodies in peripheral neuropathies	Ng, Judy King Man et al.	University of Munich	NEUROLOGY	2012	43	351
9.	Rituximab in treatment-resistant CIDP with antibodies against paranodal proteins	Querol, Luis et al.	Universitat Autònoma de BarcelonaUniversitat Autònoma de Barcelona	NEUROLOGY-NEUROIMMUNOLOGY & NEUROINFLAMMATION	2015	43	338
10.	Autoantibodies to nodal isoforms of neurofascin in chronic inflammatory demyelinating polyneuropathy	Delmont, Emilien et al.	Aix-Marseille Universite	BRAIN	2017	37	335

## Discussion

Nowadays, bibliometric analyses are increasingly used to identify the status and trends in each field. This is the first study to conduct a bibliometric analysis of AN-related literature and systematically identify scientific research trends, prolific authors, countries and institutions, key topics, and citation patterns in this field. Using the Web of Science Core Collection database, AN-related publications from the last 20 years (2005–2025) were systematically searched, and a total of 109 articles were evaluated by bibliometric analysis according to the established inclusion and exclusion criteria.

According to the results obtained, a significant increase in the number of publications was observed after 2021. This increase parallels the definition of AN disease by EAN/PNS in 2021 [6]. 2022 stood out as the most productive year with 16 publications. Although the number of publications in 2021 was lower than in 2022, it is noteworthy that the number of citations was the highest of all years. Statistically, the number of publications showed an increasing trend (R^2^ = 0,859, p < 0,001). This finding indicates an increasing academic and clinical interest in the topic. However, the fact that the number of publications is not yet at the desired level may be related to the fact that the disease was separated from the CIDP subgroup by the EAN/PNS in 2021 and studied as a new disease and is rare [20].

The results of this study also reveal the process that led to the identification of AN as a distinct clinical entity from CIDP. The most cited studies focused on antibodies to paranodal proteins such as neurofascin-155, contactin-1, and CASPR. The discovery of these antibodies and the identification of associated phenotypes were critical in distinguishing AN as a distinct disease. In particular, the study “Neurofascin IgG4 antibodies in CIDP associate with disabling tremor and poor response to IVIg” by Queol, Luis. et al. (2014) has reached the highest number of citations and had the strongest citation burst between 2015 and 2019 [32].

Some studies have reported mortality rates of up to 50%, especially in patients carrying IgG1 subclass pan-neurofascin antibodies [18]. In this subgroup, fatal outcomes are related to severe neurological complications such as respiratory failure and rapid disease progression. [33]. This highlights the importance of implementing rapid, targeted and aggressive treatment strategies in patients diagnosed with AN [18]. According to the results of the analysis, European-based institutions have a higher proportion of AN-related studies. France, Spain and Germany have a combined share of 66% in the number of publications. Furthermore, geographical and/or ethnic differences may play a role in variations in incidence [34]. Such studies may provide valuable insights into the aetiology of the disease. Although HLA associations with NF155-positive AN have been described most prominently in East Asian cohorts, Western studies have also confirmed significant links. Thus, HLA predisposition should be regarded as relevant across populations rather than region-specific. [14, 35]. However, disparities in access to diagnostic tools and specialized treatment centers may account for underrepresentation from other regions [36]. Because it is therefore plausible that the most productive institutions, Hospital de la Santa Creu I Sant Pau, Aix Marseille University and University of Würzburg, are also located in the most productive countries. The co-authorship analysis of Querol, Luis, Sommer, Claudia and Doppler, Kathrin, who are among the most active and influential authors, shows a strong collaboration between these researchers. It is consistent with the findings that these authors also work in the most productive countries and institutions. Japan stands out as both a productive country and one of the countries with the most productive authors and institutions. France, Germany, Spain and Japan have strong collaborations with other countries. In addition, the presence of researchers from different countries in the burst analysis shows that studies in this field have gained an international outlook. While most of the publications with the highest number of bursts are of European origin, the fact that Asian research has recently come to the fore shows that the geographical spread in this field is expanding.

Citation burst analyses reveal the authors and articles that have played an important role in the development of the AN scientific literature. These papers are not only the most cited, but also the ones that attracted the most attention in the literature during certain periods. In particular, the study by Querol, Luis et al. (2014) showing the association of neurofascin antibodies in CIDP patients with low response to IVIg stood out with 9.68 citation bursts between 2015 and 2019 [32]. Earlier studies such as Ng et al. (2012) and Devaux et al. (2012) were published between 2012 and 2017, while publications such as Fehmi et al. (2021) and Appeltshauser et al. (2023) have come to the fore with high citation burst in recent years [18, 33, 37]. It can be said that especially the studies with high citation burst before 2021 are guiding the definition of AN by showing the pathogenic mechanisms of paranodal antibodies and their clinical associations with CIDP. These results also show the evolution of the literature over time. Descriptive serological studies conducted in the early 2010s have been replaced in recent years by approaches such as clinical correlation, treatment response, and prognostic significance of disease subtypes. Studies on neuroimmune markers are still considered to be an academic trend. Considering that most of the available publications are case series or observational studies addressing the antibody-phenotype relationship, there is still a need for prospective randomized clinical trials. Prospective data comparing treatment response by antibody subtype are limited [17, 38].

In the thematic clustering analysis, the most striking cluster is the “pan-neurofascin antibodies” cluster with an average publication year of 2020 and 55 publications. The study with the highest centrality value in this cluster is “Neurofascin antibodies in autoimmune, genetic, and idiopathic neuropathies” [39]. This study serves as a conceptual bridge across different topics and within the cluster. This cluster also includes studies that recently experienced the highest citation burst and the most cited study of all time. Other prominent keywords in this cluster are pan-neurofascin antibodies, nodo-paranodal damage, neurofascin-155 antibodies, distinct phenotype, Ig-G1 pan-neurofascin antibodies. The prominence of these terms in the literature indicates that research topics in this field are more focused on mechanisms at the cellular and molecular level. This conceptual concentration also indicates gaps in the literature. In the keyword citation burst analysis, the terms Ranvier, polyneuropathy, neuropathy, nerve society guideline and autoimmune nodopathy were found to have strong bursts since 2007. This suggests that the topics have narrowed over time from general concepts of neuropathy to specific topics of AN.

It was found that most of the literature on AN was published in high impact factor journals. Neurology Neuroimmunology & Neuroinflammation, Neurology, Journal of The Peripheral Nervous System, Annals of Clinical and Translational Neurology, Brain, Journal of Neurology Neurosurgery and Psychiatry were found to be both the top 10 journals with the most publications on the topic and the top 10 journals with the most cited articles. The fact that most of the literature was published in journals with high impact factors and within the scope of the SCI-E shows that AN research has a strong presence in scientific platforms.

Analysis of study types revealed that the vast majority of the most influential publications were retrospective observational clinical studies. Although retrospective in nature, these works achieved high citation impact because they introduced antibody-based patient stratifications (e.g., anti-NF155, anti-CNTN1), which have directly influenced diagnostic classification and therapeutic approaches. The only prospective therapeutic series (rituximab) also attracted considerable attention despite its small sample size, underscoring the unmet clinical need for targeted treatments. In contrast, experimental animal model and neuropathology-based biopsy studies, while less frequent, provided critical mechanistic and morphological evidence linking autoantibody responses to nodal and paranodal pathology. These patterns highlight that in autoimmune nodopathies, translational relevance and novelty in patient stratification can outweigh classical evidence hierarchies in shaping the field. This observation not only explains why certain study designs dominate citation impact but also emphasizes the urgent need for larger prospective cohorts and high-quality clinical trials to consolidate the current antibody-driven classification framework. Considering that most of the 109 publications analyzed were case series, retrospective cohorts, and observational studies, there are still not enough randomized controlled trials on AN. This deficiency makes it difficult to establish AN-specific treatment algorithms. There is a clear need for prospective, multicenter studies comparing treatment response, especially according to different antibody types.

The fact that most influential studies were retrospective does not diminish their clinical impact. On the contrary, they have shaped current diagnostic practice by establishing antibody testing as a key determinant of disease classification and prognosis. Clinicians increasingly use antibody profiles to anticipate treatment response, particularly regarding IVIg resistance, and to consider early use of B-cell depleting agents such as rituximab. Autoimmune nodopathies exemplify the challenges of rare diseases: low prevalence, heterogeneous clinical expression, and limited high-level evidence. These characteristics make international collaboration, shared registries, and standardized laboratory assays indispensable. Policy makers should consider supporting cross-border reference networks to reduce diagnostic delay and ensure equitable access to emerging targeted therapies.

Our findings also highlight a translational gap: while basic science and experimental studies are numerous, their influence becomes clinically meaningful only when integrated into antibody-based stratification of patients. This underscores the need for future research agendas to explicitly link laboratory discoveries with clinical endpoints, in order to accelerate guideline development and therapeutic innovation. In addition, comprehensive epidemiological data on the distribution of AN cases by country are not yet available. This may be related to the new definition and rarity of the disease. Rare disease research networks have proven to be effective in this context by consolidating patient data and facilitating international collaboration [40]. With the planning of multicenter epidemiological studies, clearer data on the global incidence, clinical features and treatment outcomes of AN can be obtained. Studies in the field of AN are not only related to neurology, but also to immunology, nephrology and pediatrics. This emphasizes the need for interdisciplinary research in this disease. In the future, multicenter biomarker studies in neuroimmunology may revolutionize the early diagnosis and monitoring of AN.

Since studies prior to 2021 were evaluated and included with respect to the current diagnostic criteria for AN, the inclusiveness of the literature with respect to AN was increased. To our knowledge, this is the only bibliometric analysis study of AN in the literature.

This study has several limitations. First, data were collected only from WoSCC, a database widely used for bibliometric analysis and known for its timely and comprehensive updates of citation networks, so some publications may have been missed. Only English language publications were included. Publications prior to 2005 were not included in the analysis. Some recently published high quality articles may not have been adequately assessed for their contribution to the literature due to low citation counts.

## Conclusion

With the definition of AN, the disease is a current topic for researchers. The antibodies involved in the pathogenesis of the disease are still a hot topic. However, the treatment process continues to evolve. It is crucial to strengthen collaboration between countries and institutions to provide stronger evidence for treatment recommendations for AN. As this disease is already rare, it is important that future studies are as “multicentric” as possible and that long-term patient follow-up data are collected in a standardized manner. In addition, prospective study designs should be emphasized to provide more precise treatments and improve outcomes. By systematically identifying research gaps, key players, and emerging topics, this bibliometric analysis offers a valuable roadmap for future investigations and contributes to a deeper understanding of this rare but clinically significant AN.

## Data Availability

The data that support the findings of this study are available from Web of Science Core Collection but restrictions apply to the availability of these data, which were used under license for the current study, and so are not publicly available. Data are however available from the authors upon reasonable request and with permission of Web of Science Core Collection.
